# Draft genome sequences of two strains of *Pseudomonas fluorescens sensu stricto* isolated from strawberry in Japan

**DOI:** 10.1128/mra.00620-25

**Published:** 2025-08-25

**Authors:** Tomohiro Morohoshi, Nobutaka Someya

**Affiliations:** 1 Graduate School of Regional Development and Creativity, Utsunomiya University 13144, Utsunomiya, Tochigi, Japan; 2 Institute for Plant Protection, National Agriculture and Food Research Organization (NARO) 13516, Tsukuba, Ibaraki, Japan; The University of Arizona, Tucson, Arizona, USA

**Keywords:** *Pseudomonas fluorescens*

## Abstract

Here, we report the draft genome sequences of two strains of *Pseudomonas fluorescens*, MAFF 301597 and MAFF 301598, isolated from strawberry in Japan. The genome sizes of MAFF 301597 and MAFF 301598 were 6,627,466 bp with a G+C content of 60.0% and 6,449,710 bp with a G+C content of 60.1%, respectively.

## ANNOUNCEMENT

*Pseudomonas fluorescens* is a Gram-negative environmental bacterium that has been used in various studies worldwide (1). The genome sequences of approximately 200 strains of *P. fluorescens* have been deposited in the RefSeq database at NCBI (2) as of June 10, 2025. The average nucleotide identity (ANI) values between *P. fluorescens* ATCC 13525^T^, and these strains have been already calculated in the NCBI genome database (https://www.ncbi.nlm.nih.gov/datasets/genome/). However, only nine strains were identified as *P. fluorescens* based on the 95% ANI threshold. This suggests that most strains of *P. fluorescens*, which have been used worldwide, were not verified as true *P. fluorescens* (*Pseudomonas fluorescens sensu stricto*). In this study, we determined the draft genome sequences of two isolates from strawberry in Japan, which are candidates for *P. fluorescens sensu stricto*, and demonstrated that they could be classified as *P. fluorescens sensu stricto* at the genome sequence level.

MAFF 301597 and MAFF 301598 have been isolated from strawberry fruit in 1985 at Tochigi, Japan. We obtained these strains from the NARO Genebank (Tsukuba, Japan). The 16S rRNA and *rpoD* sequences of the two strains showed high identity (over 99%) with those from *P. fluorescens* ATCC 13525^T^ (GenBank accession no. LT907842). Single colonies of two strains formed on Tryptic Soy Broth (TSB; Becton, Dickinson and Co., Sparks, MD, USA) agar plates within a few passages were inoculated into TSB liquid medium and incubated overnight at 30°C. Bacterial cells were collected by centrifugation, and genomic DNA was extracted using the NucleoSpin Microbial DNA kit (Takara Bio, Shiga, Japan). Library construction and sequencing were performed using the commercial services of Bioengineering Lab. Co., Ltd. (Kanagawa, Japan). Briefly, a DNA library was constructed using the MGIEasy DNA Adapters-96 (Plate) kit, MGIEasy circularization kit, and DNBSEQ-G400RS high-throughput sequencing kit (MGI Tech Co., Ltd., Shenzhen, China). DNBSEQ 2×  200 bp paired-end sequencing was performed using a DNBSEQ-G400 sequencer (MGI Tech). Adapters and low-quality bases were trimmed from raw reads using Trimmomatic version 0.39 (3). Adapter-trimmed raw reads were assembled through the Shovill version 1.1.0 (https://github.com/tseemann/shovill) with SPAdes version 4.0.0 (4). The assemblies were annotated using the DDBJ Fast Annotation and Submission Tool (DFAST) pipeline version 1.2.16 (5). The coding sequences (CDSs) were predicted using MetaGeneAnnotator version 2008/08/19 (6). Genes coding for tRNA and rRNA were identified using Aragorn version 1.2.38 (7) and Barrnap version 0.8 (https://github.com/tseemann/barrnap), respectively. MetaGeneAnnotator, Aragorn, and Barrnap were chosen as parameters within the DFAST pipeline. A summary of the genome sequence analysis is shown in Table 1. Default parameters were used for all software, unless otherwise specified.

**TABLE 1:** Genomic information of the two strains of *P. fluorescens* isolated from strawberry in Japan

Genome characteristics	Strains	
	MAFF 301597	MAFF 301598
Paired-end reads	5,629,544	5,748,741
Total DNA sequenced (bp)	2,251,817,600	2,299,496,400
Genome size (bp)	6,627,466	6,449,710
Coverage (×)	340	357
Contigs	59	66
CDSs	6,008	5,784
N50 (bp)	530,293	496,308
GC content (%)	60.0	60.1
BioProject accession no.	PRJDB23648	PRJDB23648
BioSample accession no.	SAMD00919759	SAMD00919760
DRA accession no.	DRR687326	DRR687327
DDBJ/ENA/GenBank accession no.	BAAHPZ000000000	BAAHQA000000000
ANI value with ATCC 13525^T^ (%)	99.40	99.39
16S rRNA and *rpoD* sequences	NARO Genebank database	NARO Genebank database

The whole genome similarity between the two strains and *P. fluorescens* ATCC 13525^T^ was analyzed by calculating the average nucleotide identity (ANI). ANI values were calculated using the pairwise calculation method with the OrthoANIu online tool (https://www.ezbiocloud.net/tools/ani) (8). As the ANI values of both strains with *P. fluorescens* type strain NCTC 10038^T^ were higher than 99% and significantly exceeded the threshold of 95% (Table 1), the two strains could be classified as *P. fluorescens sensu stricto*.
